# Mercury in the Soil of Two Contrasting Watersheds in the Eastern United States

**DOI:** 10.1371/journal.pone.0086855

**Published:** 2014-02-14

**Authors:** Douglas A. Burns, Laurel G. Woodruff, Paul M. Bradley, William F. Cannon

**Affiliations:** 1 United States Geological Survey, Troy, New York, United States of America; 2 United States Geological Survey, Mounds View, Minnesota, United States of America; 3 United States Geological Survey, Columbia, South Carolina, United States of America; 4 United States Geological Survey, Reston, Virginia, United States of America; Uppsala University, Sweden

## Abstract

Soil represents the largest store of mercury (Hg) in terrestrial ecosystems, and further study of the factors associated with soil Hg storage is needed to address concerns about the magnitude and persistence of global environmental Hg bioaccumulation. To address this need, we compared total Hg and methyl Hg concentrations and stores in the soil of different landscapes in two watersheds in different geographic settings with similar and relatively high methyl Hg concentrations in surface waters and biota, Fishing Brook, Adirondack Mountains, New York, and McTier Creek, Coastal Plain, South Carolina. Median total Hg concentrations and stores in organic and mineral soil samples were three-fold greater at Fishing Brook than at McTier Creek. Similarly, median methyl Hg concentrations were about two-fold greater in Fishing Brook soil than in McTier Creek soil, but this difference was significant only for mineral soil samples, and methyl Hg stores were not significantly different among these watersheds. In contrast, the methyl Hg/total Hg ratio was significantly greater at McTier Creek suggesting greater climate-driven methylation efficiency in the Coastal Plain soil than that of the Adirondack Mountains. The Adirondack soil had eight-fold greater soil organic matter than that of the Coastal Plain, consistent with greater total Hg stores in the northern soil, but soil organic matter – total Hg relations differed among the sites. A strong linear relation was evident at McTier Creek (r^2^ = 0.68; p<0.001), but a linear relation at Fishing Brook was weak (r^2^ = 0.13; p<0.001) and highly variable across the soil organic matter content range, suggesting excess Hg binding capacity in the Adirondack soil. These results suggest greater total Hg turnover time in Adirondack soil than that of the Coastal Plain, and that future declines in stream water Hg concentrations driven by declines in atmospheric Hg deposition will be more gradual and prolonged in the Adirondacks.

## Introduction

Soil is the largest reservoir of mercury (Hg) storage in global terrestrial ecosystems, and transfers to and from soil are pivotal in Hg cycling among vegetation, the atmosphere, groundwater, surface water, and the oceans [1]. A recent study estimates the global soil pool size (year 2000) at 240,000 Mg Hg, and indicates that Hg storage has increased by about 20% since 1840 largely due to atmospheric Hg deposition primarily derived from anthropogenic emissions sources such as coal burning, cement manufacturing, and other industrial and mining activities [2]. Soil Hg concentrations vary widely from 10 ng/g to 1000 ng/g in rural and remote areas, and from 100 ng/g to >10,000 ng/g in urban, industrial, and mineralized/mined lands [3], [4]. This Hg is largely bound to soil organic matter (SOM), and Hg concentrations are typically strongly related to measures of SOM or soil organic carbon (SOC) [5]–[7]. Under oxidized conditions, Hg (II) is strongly bound to organic thiol groups, which are typically present in excess of available Hg in most soil [8], [9]. Under anoxic conditions, meta-cinnabar and other sulfide minerals may also be important Hg forms [10], though the presence and abundance of these mineral forms in soil without a metal sulfide ore genesis is not clear [11]. Additionally, Hg may be adsorbed either specifically or non-specifically to SOM and to mineral surfaces such as iron and aluminum oxy-hydroxides [12].

Given the strong affinity of Hg for SOM, soil Hg concentrations are typically greatest where SOM is concentrated, in shallow O- or A-horizons, and these concentrations typically decrease with depth [7]. The O-horizon commonly has Hg concentrations an order of magnitude greater than that of the mineral-dominated B-horizon, though Hg/SOM is typically greater in the B horizon [7], [13], [14]. Similarly, organic-rich soil such as peat typically has greater Hg concentrations than those of soil dominated by inorganic material [6]. Soil in areas with high geogenic Hg concentrations can show the reverse of the typical depth pattern, with greater Hg concentrations in the deep soil than near the surface [15]. Despite the greater Hg concentrations commonly measured in surface horizons, deeper mineral horizons generally store more Hg because of the much greater mass of mineral-dominated subsoil in most landscapes [4], [6], [16]. Total soil storage of Hg can vary from a few mg/m^2^ to >50 mg/m^2^, and these values vary, similarly to those of Hg concentrations, as a function of source loads of atmospheric Hg deposition, the amount of SOM in soil, and geogenic concentrations in parent material [3], [6]. Disturbance factors related to fire history, agriculture, forest harvesting, mining, and other human land uses also affect Hg concentrations and storage in soil [17], [18].

Several studies have described strong relations between soil Hg concentrations and SOM/SOC, consistent with the strong affinity of Hg for binding with thiol groups [5], [7], [13], [19]. The slopes and intercepts of these Hg–SOM relations vary greatly, however, as a function of regional location, horizon and/or depth, landscape type, and vegetation type [6], [16], a finding that is expected given the heterogeneity of SOM and the excess of thiol-like Hg binding sites generally available [9]. Some study results are consistent with variation of Hg/SOM as a function of the rate of atmospheric Hg deposition [5], [15], although variation in geogenic Hg may be a contributing factor as well [20].

Although measurements of total Hg (THg) in soil provide important data necessary to better understand the Hg cycle, data on methyl Hg (MeHg) concentrations in soil are more pivotal to understanding bioaccumulation in terrestrial and aquatic food webs. The ratio of MeHg/THg varies widely in soil from <1% to >20% [4], [6], [8]. Wetland soil generally has greater MeHg concentrations and MeHg/THg than soil in steeper upland parts of landscapes, and more complex patterns are often superimposed on this general gradient. One study in a boreal forest catchment in Sweden found greater MeHg concentrations and MeHg/THg in soil at stream bank and near-stream locations than 20 m from the stream [8]. Another more recent investigation showed the highest MeHg concentrations in soil pore water where upland soil borders peaty wetland soil suggesting that the distal riparian area may have greater soil MeHg concentrations than the near-stream riparian area [21]. The MeHg content of soil is important because of the potential for mobilization into soil solution followed by surface and/or subsurface runoff and transport to local surface waters where bioaccumulation in aquatic food webs may then occur.

Despite many published studies on Hg in soil [3]–[7], little is known about controls on the relation of Hg concentrations and stores in soil to those of adjacent surface waters. Elevated Hg levels are often measured in surface waters with extensive wetlands where high THg and MeHg concentrations and storage in soil have been demonstrated, such as lake watersheds in the US Upper Midwest [22] and the Adirondacks of New York [16].

In this study, we compare Hg concentrations and storage in the soil of two watersheds, located in “hot spot” regions where Hg in predator fish and/or aquatic-feeding birds consistently exceed thresholds for human health warnings and for adverse effects on wildlife [23], [24], the Adirondack Mountains of New York, and the Coastal Plain of South Carolina [25], [26]. Soil THg and MeHg concentrations and storage are compared across three different landscape types in each watershed, uplands, riparian areas, and wetlands, because hydrologic/transport dynamics among these landscapes are important in mobilization of Hg to local streams and subsequent bioaccumulation in the aquatic ecosystem [25]–[27]. Our objectives are to describe how Hg concentrations and pool sizes in soil differ between these two watersheds and among different landscape types, and to discuss the implications of any differences that can be readily identified.

### Study Sites

Soil samples were collected in two geographic locations: (1) Fishing Brook watershed in the Adirondack Mountains of New York, and (2) McTier Creek watershed in the Coastal Plain of South Carolina. A brief description of these study sites follows with an emphasis on soil; however, the reader is referred to [28] for more detailed descriptions of these study areas.

Fishing Brook (FB) is a 65.6 km^2^ watershed in the westernmost headwaters of the Hudson River basin. The watershed is a heterogeneous landscape characterized by forested uplands, riparian floodplains, and scattered open water bodies. The watershed is dominantly forested (86.3%) by Adirondack Northern Hardwood Forest [29]. Sugar maple (*Acer saccharum*), yellow birch (*Betula alleghaniensis*) and American beech (*Fagus grandifolia*) are the dominant tree species below 980 m elevation, and balsam fir (*Abies balsamea*) and red spruce (*Picea rubens*) are the dominant species above this elevation [29]. Additionally, balsam fir and red spruce are dominant in wet soil at the base of hillslopes and in riparian areas adjacent to streams and ponds. The watershed consists of 8.2% wetland area (National Wetlands Inventory (NWI) approach, [30]), and scrub-shrub is the dominant wetland type, with speckled alder (*Alnus incana*) a dominant species [31]. Open water is 2.7% of watershed area, with numerous small ponds and lakes. Only 0.7% of the watershed is considered developed, as low density residential land use, some dirt roads, and one highway (Rte. 28N).

Upland soils in the FB watershed are mainly Spodosols developed in coarse, bouldery glacial till derived from the Precambrian metamorphic bedrock of the region [32]. The most common soil types found in the watershed include the Skerry, Becket, Adirondack, and Tunbridge Soil Series, generally well-drained, bouldery sandy loams classified as Haplorthods [33]. The most common soil types in riparian wetland areas are broadly classified as Entisols or Histosols and include the Wonsqueak, Rumney, and Bucksport Soil Series, mixtures of loamy sand or silt, muck, and peat with a water table that ranges from just below land surface during the growing season, to near or above the land surface during the dormant season [33]. These riparian soil types are generally developed in fluvial or glaciofluvial sediments, and thus are not as rich in organic matter as the deep peat riparian wetlands in the western Adirondacks described in [34].

McTier Creek (MC) is a 79.4 km^2^ watershed (defined by the USGS stream gage near New Holland, SC) tributary to the Edisto River, and located in the Inner Coastal Plain. The watershed consists of 49.6% forested land cover, of which loblolly pine (*Pinus taeda*) is most common. About 6.4% of the watershed is wetland area (NWI approach, [35]), and bottomland hardwoods such as black tupelo (*Nyssa sylvatica*) are dominant in floodplain areas where these wetlands are generally found. Additionally, 1.2% of the watershed consists of open water, 14.9% is agricultural land, and 4.7% is developed [28]. The remaining land cover is mainly herbaceous upland.

Soil at MC is developed in sandy fluviomarine deposits and is generally classified as an Ultisol on ridges and hillslopes and as an Entisol or Inceptisol in riparian floodplains [36]. The Troup, Vaucluse, and Ailey Series are those most common on ridges and hillslopes, generally an excessively drained sandy soil with clay-rich kaolinitic deposits found at depth in isolated areas [36]. Floodplains are dominated by the poorly drained Bibb Series loamy sand and Johnston Series mucky loam, the latter of which sometimes has standing water at the surface signifying wetlands [36].

A comparison of the landscape and soil of these two watersheds reveals some similarities and some sharp differences. Both watersheds are dominated by upland forests, although the FB landscape is generally steeper (median slope = 12.5%) than that of MC (median slope = 5.4%), and MC also includes almost 20% developed land and agriculture, whereas FB has <1% developed land. Narrow floodplains dominated by sandy and loamy deposits as well as some mucky or peaty soil are prevalent in both watersheds, and riparian wetlands are commonly found in these floodplains. Riparian area defined as all land within an elevation of 0.65 m of the stream network [37] is 14.1% of the MC watershed area and 18.4% of the FB watershed area. When all wetland area located within this riparian delineation is excluded from these calculations, these riparian area values decrease to 9.3% of the MC watershed area and 12.3% of the FB watershed area. The two watersheds also have similar proportions of wetland area with a slightly greater value in the FB drainage (8.2%) than in the MC drainage (6.4%) according to NWI methods [35]. This difference narrows further (FB = 9.3%, MC = 8.2%) according to the wetland classification approach of the National Land Cover 2001 Dataset [38], but we apply NWI data in the current paper.

Perhaps the greatest difference between these two watersheds is their respective climates. FB experiences a cold, humid continental climate with mean annual air temperature of 4.9°C and mean annual precipitation of 1074 mm (Newcomb, NY, 1981–2010, [39]). Mean minimum daily air temperature is <0°C for >6 months of the year, much of the winter precipitation falls as snow, and spring snowmelt is a major feature of the annual hydrograph [40]. In contrast, MC experiences a subtropical moist climate with mean annual air temperature of 17.9°C and mean annual precipitation of 1276 mm, of which little falls as snow (Aiken, SC, 1981–2010, [41]). Stream runoff also differs greatly among these two regions. For example, the Edisto River near Givhans, SC of which MC is tributary has mean annual runoff of 273 mm (1981–2010, [42]), whereas the Hudson River at North Creek, NY of which FB is a tributary has mean annual runoff of 735 mm over the same period [42], a difference that is largely attributed to the role of snowmelt in New York, and to higher annual rates of evapotranspiration in South Carolina than in New York.

## Materials and Methods

### Field

**Figure 1:**
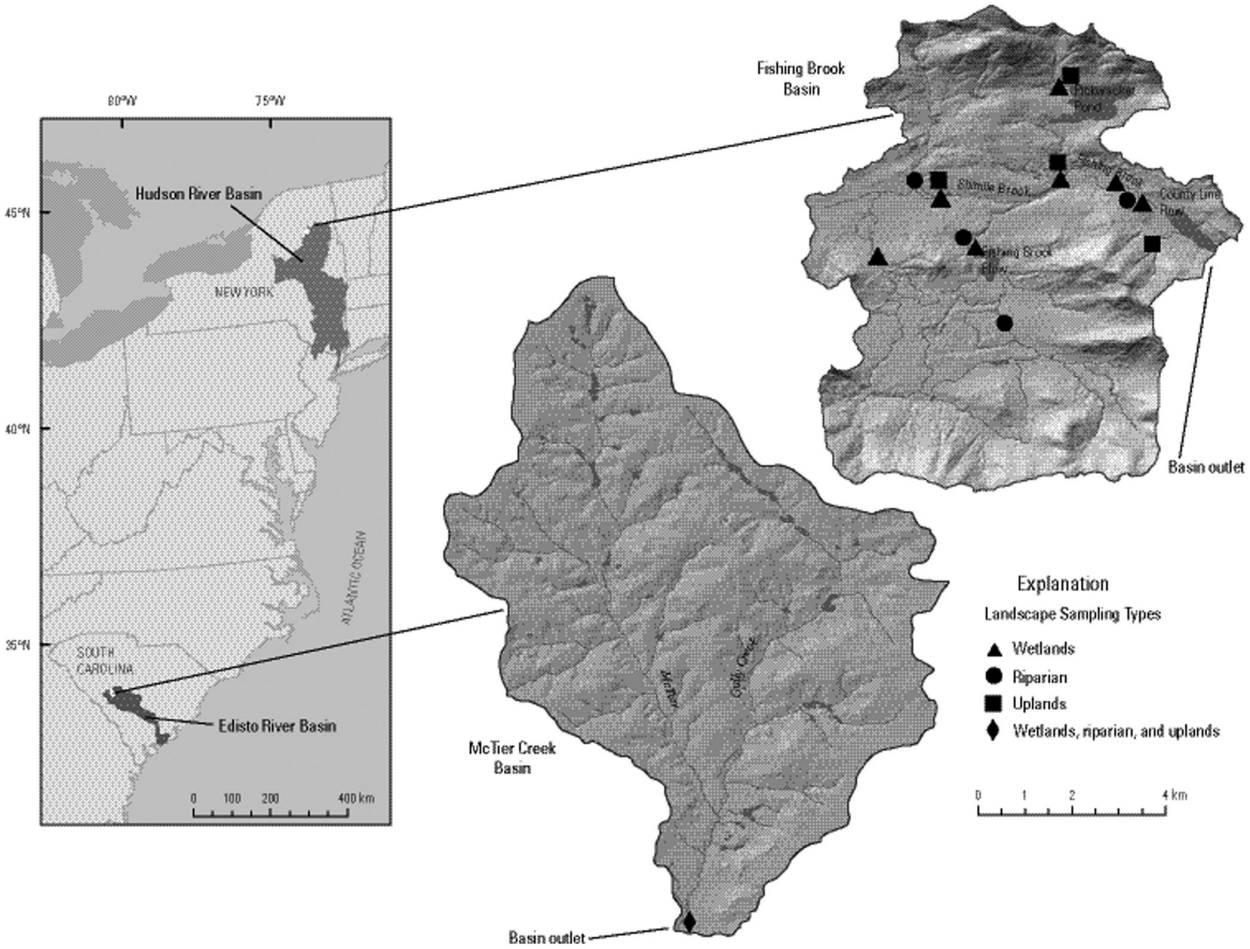
Study site maps. Locations of McTier Creek watershed within the Edisto River basin, and Fishing Brook within the Hudson River basin. Symbols indicate approximate locations where soil samples were collected in different landscape types as described in text. Note that only one symbol appears in the McTier Creek map because the 24 sampling sites were located too close together to be resolved in a map of the whole watershed.

Soil sampling focused on the key landscape types in each watershed. At FB, samples were collected from upland hillslope areas in northern hardwood forest, along riparian areas in spruce-fir forest, and in wetlands [43]. A total of 163 soil samples were collected at 70 distinct locations during Sept. 2008 (Fig. 1). Sampling sites were on private land owned by Finch Pruyn Company (now Finch Company), and verbal permission was provided for collecting environmental samples including a key to access locked gates needed to reach many of the sites. At MC, samples were collected along two transects from upland hilltops and hillslopes, riparian floodplains, and wetlands [43]. A total of 81 samples were collected from 24 distinct locations in Nov. 2008 (Fig. 1). Sampling sites were on private land owned by the family of the late Senator Strom Thurmond who provided verbal permission for access and the collection of environmental samples. For the purposes of this analysis, soil data are distinguished for uplands, riparian floodplains, and wetlands in each watershed.

Samples were collected with a shovel and hand auger and placed into sediment vials immediately upon collection. Nitrile gloves were used when handling soil to avoid contamination. At least two depths/horizons were sampled at each location, either an Oa (FB) or A (MC) horizon, and a B horizon. The B horizon represents a depth integrated sample that reflects the complete horizon to a depth of 40 cm; sampling did not attempt to make fine scale distinctions within the B horizon. At a few of the sites, samples were collected deeper in the profile, either the B/C or C horizon; E-horizon samples were also collected at both Fishing Brook and McTier. In wetland areas without well-developed soil horizons, samples were collected by depth interval. In such soil, the top 10 cm was typically equated to an Oa or A horizon for the purposes of this data analysis, and a second interval below this depth, typically 10 cm to about 20 to 40 cm, was equated to a B horizon for the purposes of this data analysis.

### Laboratory

Oa horizon samples were air dried, milled, and split. A portion was set aside for Hg analysis, and the remainder was heated in an oven at 500°C for 13 hr to determine loss-on-ignition. For mineral soil horizons, samples were air dried and sieved to <2 mm. Total carbon (C) in mineral-dominated soil samples was determined by infrared detection of CO_2_ in an automated C analyzer [44], carbonate C was determined by coulometric titration [45], and organic C was determined by difference. Organic C data were multiplied by 1.9 to convert these values to percent organic matter for the purposes of data analysis [46].

Mercury was analyzed by cold vapor atomic absorption spectrometry according to a method described in Smith et al. [47]. This method is a modification of U.S. Environmental Protection Agency Method 7471B [48], and is considered an analysis of total Hg. The method detection limit is 10 ng/g. A lab-homogenized sub-sample of soil that was frozen as soon as possible after collection was shipped to the laboratory on dry ice, and held frozen until analysis of MeHg by cold vapor atomic fluorescence spectrometry. Details of the MeHg analysis method, which has a 0.08 ng/g detection limit, are described in [49].

Laboratory quality assurance and quality control procedures for the analytical methods used in this study are described in [43] and [47], except for those of MeHg, which are described in [49]. Sixteen duplicate samples were collected in the FB watershed, and 7 were collected in the MC watershed. Precision for FB duplicates samples as represented by the mean standard deviation and mean relative standard deviation (in parentheses) of these duplicate analyses was 4.9 ng/g (8.4%) for total Hg, and 7.4% (22.0%) for percent organic matter. Precision for MC duplicates was 10 ng/g (21.1%) for total Hg, and 0.38% (12.4%) for percent organic matter. Duplicate analyses were not available for MeHg, but analytical precision data for multiple analyses of a reference solid sample indicate a relative standard deviation of 8.6% for the method used in this study [49].

Soil data used in this paper are available in [43], and can be freely downloaded.

### Storage Estimates

THg and MeHg stores in soil were estimated at each study site. Estimates of soil element stores have high uncertainty due to a variety of factors including the presence of rocks, variations in the depth and thickness of soil horizons, high spatial heterogeneity of chemical and physical variables, and difficulties in accurately measuring bulk density, among others [50]. These sources of uncertainty should be carefully considered when comparing estimates of soil element stores among various studies in which assumptions can vary widely. In this investigation, we did not attempt to quantify soil rock content, a major source of uncertainty in element analyses [51], particularly in formerly glaciated terrain overlain by coarse till such as the FB watershed (rocks were not evident in the MC watershed soil). We developed estimates of THg and MeHg for the uppermost 40 cm of soil at each site, excluding fresh and partially decomposed litter (considered Oi and Oe horizons in some soil types), which was not sampled. These storage estimates were developed for the uplands, riparian area, and wetlands in each watershed using soil samples collected in each of these landscape types.

In Fishing Brook, the Oa horizon was assumed to be the uppermost 5 cm of soil in upland areas, the E horizon the next 5 cm, and the B horizon was assumed to include a depth interval of 10 to 40 cm, broadly consistent with observations in these Spodosols [33] and representing the mean of depth interval measurements made at the time of sampling. In the Entisol and Histosol soils that were dominant in riparian and wetland areas at FB and that frequently had poorly developed horizons, Hg data from Oa samples were applied to the depth interval of 0 to 10 cm, and B-horizon Hg data were applied to a depth interval of 10 to 40 cm.

In MC, the A horizon Hg data were applied to a depth interval of 0 to 4 cm in upland areas, 0 to 11 cm in riparian areas, and 0 to 20 cm in wetland areas based on the mean of depth measurements in each of these landscapes at the time of sampling. The B horizon Hg data were applied to the depth interval from the bottom of the A to 40 cm. The South Carolina soil survey typically includes an E horizon below the A in the soil types that dominate in uplands, and a C or more extensive A horizon below our A horizon classification in the soil types that dominate in riparian and wetland areas [36]. However, we recognize that some samples that were included in the B horizon category may actually be A, E, or C horizons samples based on this classification.

Bulk density was measured at 15 sites in A horizon soil at MC and at 38 sites in B horizon soil at FB. These measurements were made by sampling a known volume of soil and weighing this sample after drying. These measurements did not cover the full range of organic matter content measured in soil at these sites. In the case of MC, low organic matter soil (<5%) was not represented, whereas at FB, high organic matter soil (>24%) was not represented. Available bulk density measurements were averaged within 5 categories each representing a 5% range of soil organic matter (SOM) from 0% to 25% at FB, and at MC were averaged and placed into 4 categories each representing a 10% range up to 40% SOM (first category was 5% to 10%). These categorical data were best fit by a highly significant (p<0.001) power law regression of the form: bulk density = a * percent organic matter (−exp b). These equations were used to calculate bulk density values in the range of SOM >5% at MC and SOM<24% at FB. This approach provided good estimates of bulk density as demonstrated by mean deviations from measured values of +0.016 g/cm^3^ at FB and −0.004 g/cm^3^ at MC with no evident bias (organic matter vs. residuals linear regression, slope not significantly different than 0, p>0.10) across the OM range. For the SOM ranges of 0–5% at McTier Creek and 24–100% at Fishing Brook, a pedo-transfer equation was applied [52]. This approach is based on a simple mixing model between the bulk density values of 100% organic matter and that of 100% mineral soil and has proven useful in many previous investigations [53], [54]. The value used in this equation for 100% SOM at MC was 0.10 g/cm^3^ derived from the power law regression, and the value for 100% mineral soil was 1.70 g/cm^3^ based on sandy Coastal Plain B-horizon soil with little organic matter [55], [56]. The value used in this equation for 100% SOM at FB was 0.11 g/cm^3^ based on data from Spodosols in the northeastern US [57] and the value used for 100% mineral soil was 1.99 based on the power law regression equation as it approaches 0% SOM (at 1% SOM).

### Statistical Analyses

Seven of 163 soil samples collected at Fishing Brook had THg concentrations less than the 0.1 ng/g laboratory detection limit, and 6 of 81 soil samples collected at McTier Creek also had THg concentrations less than this detection limit. Therefore, non-parametric statistical analyses appropriate for censored data were used for any calculations that included THg data. Although there were no censored MeHg values, non-parametric statistical analyses were applied to these data as well because data were not normally distributed. The Mann-Whitney Rank-Sum test [58] was applied for pairwise comparisons, and the Kruskal-Wallis test for multiple comparisons [59]. Maximum likelihood regression was used instead of least squares linear regression, and a likelihood r^2^ value calculated for each model [60]. Finally, relations among variables were determined by Spearman rank correlation analysis. All statistical analyses were performed with SigmaStat, part of the SigmaPlot software (v. 12, use of product names in this paper is for identification purposes only and does not imply endorsement by the U.S. Government), except for maximum likelihood regression, which was performed with SAS.

## Results

THg and MeHg concentrations and the THg/MeHg ratio for the two watersheds are compared first in Oa/A horizon samples and second in B horizon samples. These soil concentrations and ratios are also compared across landscape type within each watershed. The relations of Hg species concentrations to percent SOM are then presented. Finally, estimated storage of THg and MeHg in soil is presented and compared among the two watersheds.

### Comparison of Soil Hg among Watersheds – Oa and A Horizon Soil

**Figure 2:**
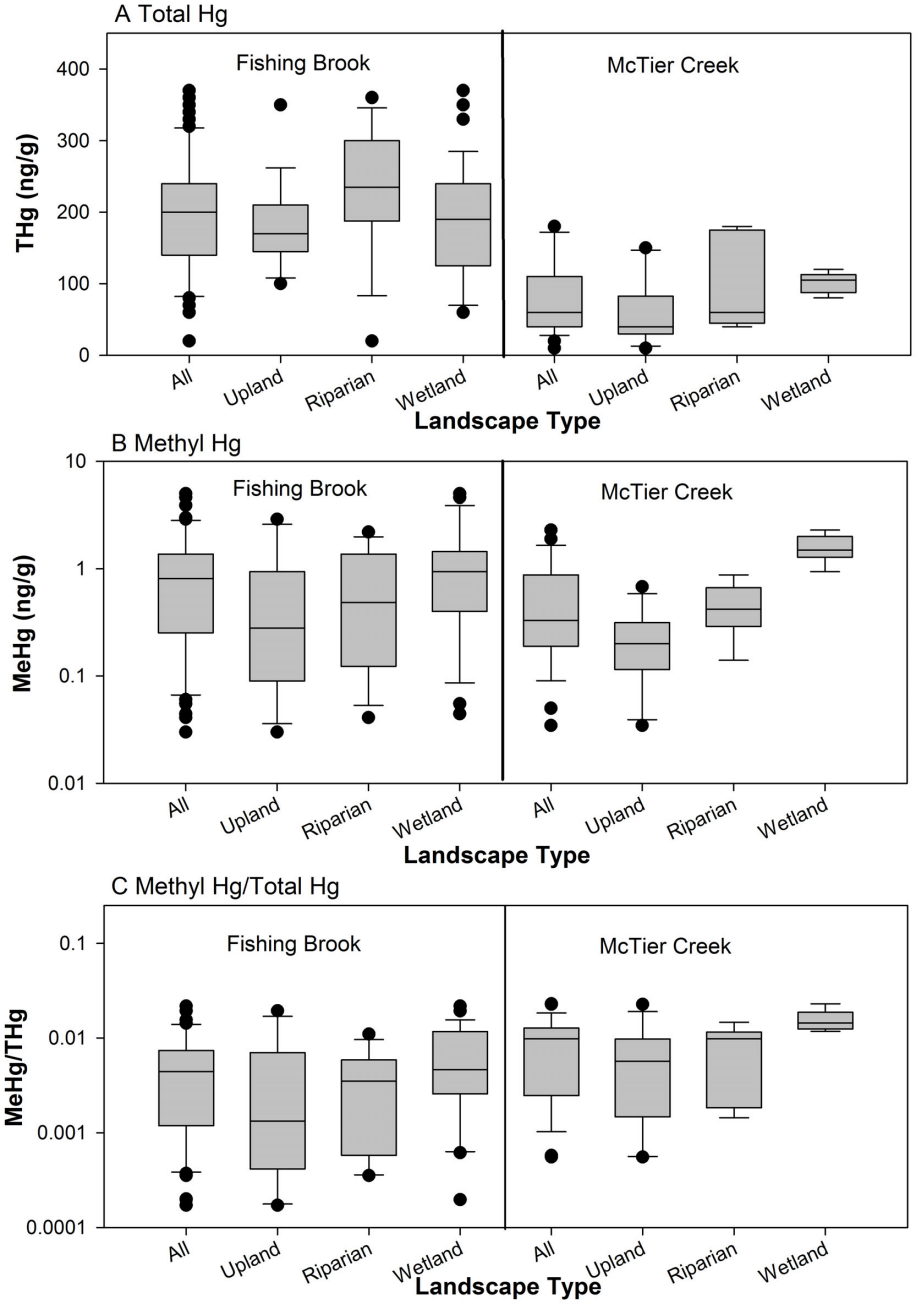
Mercury values in soil Oa/A horizon samples. Box plots of soil Hg concentrations measured in the Fishing Brook, NY and McTier Creek, SC watersheds for samples collected from Oa/A horizons. A Total Hg, B Methyl Hg, and C Methyl Hg/Total Hg.

THg concentrations were significantly greater (p<0.05) in Oa/A horizon soil samples from FB than those from MC (Fig. 2A, Table 1). FB soil also had significantly greater THg concentrations than that of MC across all three landscape types. The median THg concentration of 200 ng/g at FB was more than three-fold greater than the median value of 60 ng/g at MC, and an approximate two-fold to four-fold difference was measured across the three landscape types. The differences in THg among soil from these two watersheds were so great, that the highest concentrations measured at MC were less than the median concentrations at FB for all samples as well as for those collected in each landscape type (Fig. 2A).

**Table 1:** Median values of THg, MeHg, and MeHg/THg in Oa/A horizon soil samples from Fishing Brook, NY and McTier Creek, SC.

Constituent	Fishing Brook				McTier Creek			
	All	Upland	Riparian	Wetland	All	Upland	Riparian	Wetland
***THg*** *(ng/g)*								
n	71	17	16	38	27	12	9	6
median	**200**	**170**	**235**	**190**	60	40	60	105
***MeHg*** * (ng/g)*								
n	52	11	12	29	27	12	9	6
median	0.81	0.28	0.48	0.94	0.33	0.20	0.42	1.50
***MeHg/THg*** * (%)*								
n	52	11	12	29	27	12	9	6
median	0.44	0.13	0.35	0.46	**0.98**	0.57	0.98	**1.40**

Note – bold font indicates median value was significantly greater (p<0.05) in site-to-site Mann-Whitney pairwise comparison tests done for all samples collected and for each landscape type.

The median MeHg concentration of 0.81 ng/g for all FB soil samples was more than two-fold greater than the median value of 0.33 ng/g for all MC samples, but the difference between these watersheds was not significant (p = 0.133; Table 1). The median value for upland and riparian samples was also slightly greater at FB than MC, but these differences were similarly not significant. In contrast, the median MeHg concentration of wetland soil samples collected at MC of 1.5 ng/g was greater than the value of 0.94 ng/g in FB wetland samples, a difference of marginal statistical significance (p = 0.088). There were fewer significant site differences for MeHg than for THg because of greater variation in the MeHg data (Fig. 2b); relative standard deviation values were 40.0% and 63.8% for THg at FB and MC, respectively, and increased to 109.4% and 99.5% for MeHg at these watersheds.

The MeHg/THg ratio showed the opposite pattern of the individual constituents with values two- to four-fold greater at MC than at FB (Table 1). This ratio was significantly greater for all samples and for those from the wetlands, but differences were not significant for riparian (p = 0.110) and upland (p = 0.148) soil. Though MeHg soil concentrations in the Coastal Plain watershed were generally less than those in the Adirondack watershed, MeHg/THg values were generally greater in the south.

### Comparison of Soil Hg between Watersheds – B Horizon Soil

**Figure 3:**
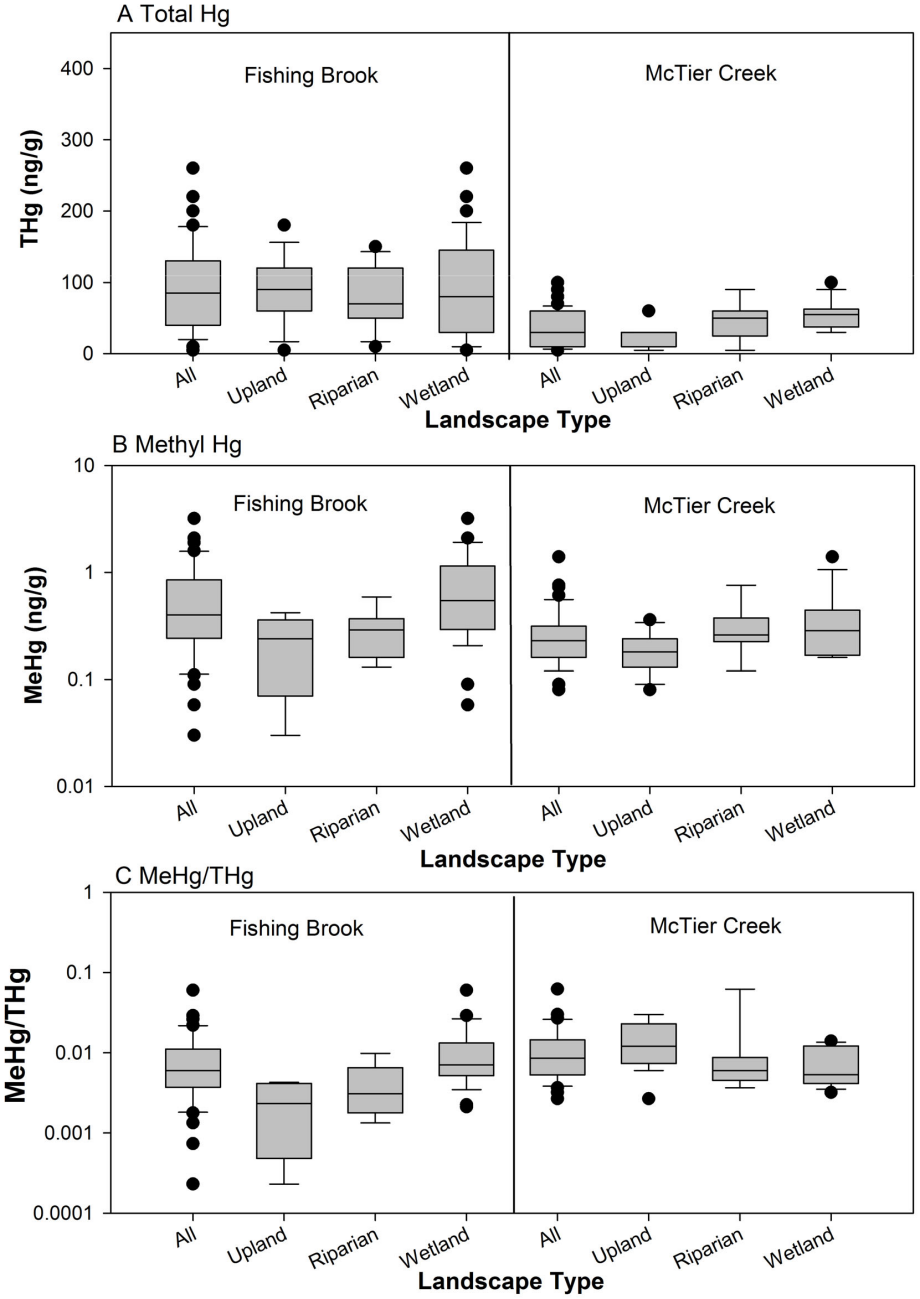
Mercury values in soil B horizon samples. Box plots of soil Hg concentrations measured in the Fishing Brook, NY and McTier Creek, SC watersheds for samples collected from B horizons. A Total Hg, B Methyl Hg, and C Methyl Hg/Total Hg.

Soil Hg concentrations were generally less in B horizon soil than those in Oa/A horizon soil at both watersheds (Table 2, Figs. 2 and 3). The inter-watershed differences in Hg concentrations in the deeper B horizon soil were broadly consistent with the patterns in the shallower Oa/A horizon soil with some minor differences. THg concentrations were significantly greater in FB B horizon soil than those of MC for all samples and for those from uplands and riparian areas, but not for wetland samples. Median MeHg concentrations in B horizon samples were generally greater at FB than at MC as in Oa/A horizon samples. Whereas these inter-watershed differences were not statistically significant in Oa/A horizon soil, the differences were highly significant for all B horizon samples (p<0.001) and significant for wetland samples (p = 0.023), but not significant for those from uplands and riparian floodplains. Finally, MeHg/THg was also generally greater in B horizon soil at MC than at FB consistent with the findings for the shallow soil horizons. These differences were highly significant for upland soil (p = 0.001), of marginal significance for all soil samples (p = 0.052), but not significant for riparian and wetland samples.

**Table 2:** Median values of THg, MeHg, and MeHg/THg in B horizon soil samples from Fishing Brook, NY and McTier Creek, SC.

Constituent	Fishing Brook				McTier Creek			
	*All*	Upland	Riparian	Wetland	All	Upland	Riparian	Wetland
***THg*** *(ng/g)*								
n	70	17	16	37	42	19	9	14
median	**85**	**90**	70	80	30	10	50	55
***MeHg*** * (ng/g)*								
n	40	5	7	28	42	19	9	14
median	**0.40**	0.24	0.29	**0.54**	0.23	0.18	0.26	0.28
***MeHg/THg*** * (%)*								
n	40	5	7	28	42	19	9	14
median	0.60	0.23	0.31	0.71	0.86	**1.20**	0.60	0.53

Note – bold font indicates median value was significantly greater (p<0.05) in site-to-site Mann-Whitney pairwise comparison tests done for all samples collected and for each landscape type.

### Comparison of Soil Hg across Landscape Types

Within the FB watershed, THg concentrations in soil were similar across all landscape types for both Oa/A and B horizon samples (Figs. 2 and 3). MeHg and MeHg/THg values were also similar and statistically indistinguishable across all landscape types for Oa/A horizon soil samples. However, in B horizon samples, MeHg concentrations and MeHg/THg were significantly different across the three landscape types. Pairwise comparisons among the landscape types showed that wetlands>riparian areas = uplands, a consistent finding for both MeHg concentrations and MeHg/THg.

MC showed more differences in soil Hg concentrations as a function of landscape type than did FB (Figs. 2 and 3). All Hg measures were significantly different among the three landscape types in both Oa/A and B horizon samples. In pairwise comparisons, wetland values were nearly always significantly greater than those of the uplands, and the riparian and upland landscape types were most often statistically indistinguishable, despite median THg and MeHg values that were greater in riparian samples. The exception to these generalizations was MeHg/THg in the B horizon samples for which the upland ratio was significantly greater than the wetland ratio, whereas the other pairwise comparisons showed no significant differences.

### Relations of Hg to Percent Soil Organic Matter

**Figure 4:**
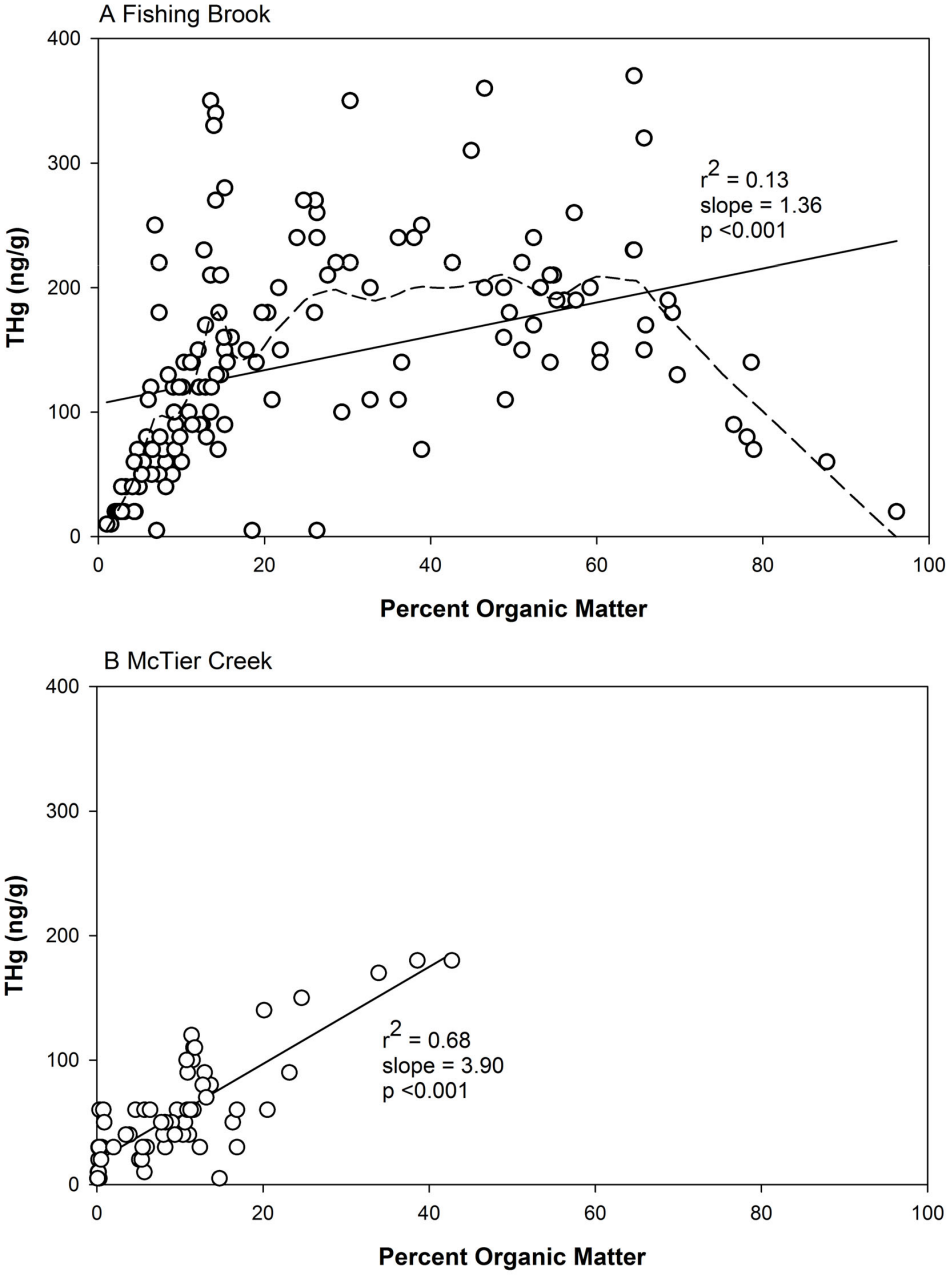
Total Hg and percent organic matter for all soil samples collected in each watershed. Solid line in each panel indicates a significant linear regression relation with r^2^, slope, and p value. A Fishing Brook. Dashed line is a Loess smooth obtained by a first order polynomial fitted to a 20% moving window of the data. B McTier Creek.

Some strong differences are evident in the relations of THg to percent SOM among these two watersheds and as a function of soil depth and landscape type (Fig. 4; Table 3). A highly significant (p<0.001) positive maximum likelihood regression relation was evident between THg and SOM for all soil samples collected at FB, but this relation explains only 13% of the variation in these data. At MC, an equally highly significant relation (p<0.001) explains 68% of the variation in these data (Fig. 4). Despite the significance of the regression relation at Fishing Brook, a LOESS smooth better fits the data pattern and confirms a generally non-linear THg-SOM relation that is highly variable throughout the range of SOM data; a generally linear increase in THg to an SOM value of about 15% (r^2^ = 0.46, p<0.001), little change and much scatter in the range of 15% to about 60%, and a linear decrease in THg at SOM values >60% (Fig. 4A). In contrast, THg values at MC show much scatter at SOM values <20%, but generally increase with SOM at values >20%, though there are few samples with high SOM values (Fig. 4B). These data highlight the generally greater SOM in FB soil where values >90% were measured, whereas the greatest SOM values in soil samples at MC were about 40%.

**Table 3:** Spearman rank correlations of THg and percent organic matter and MeHg and percent organic matter in Oa/A horizon and B horizon soil at Fishing Brook, NY and McTier Creek, SC.

Site/Landscape Position	Oa/A Horizon				B Horizon			
	THg		MeHg		THg		MeHg	
	n	r_s_	n	r_s_	n	r_s_	n	r_s_
***Fishing Brook***								
All	71	**−0.315**	52	**−**0.117	70	**0.759**	40	**0.413**
Upland	17	**−**0.129	11	0.036	17	**0.780**	5	**−**0.600
Riparian	16	0.093	12	**−**0.14	16	**0.793**	7	**−**0.071
Wetland	38	**−0.402**	29	**−**0.205	37	**0.734**	28	**0.516**
***McTier Creek***								
All	27	**0.654**	27	**−**0.125	42	**0.750**	42	**0.584**
Upland	12	**0.864**	12	**−**0.070	19	**0.694**	19	0.312
Riparian	9	**0.906**	9	**−**0.460	9	0.252	9	0.429
Wetland	6	**−**0.232	6	**−**0.377	14	**0.648**	14	0.460

Note – bold font indicates that THg – SOM or MeHg – SOM relation was significant.

The inverse relation at FB of THg to SOM at high SOM values (as per LOESS smooth) results largely from the Oa/A horizon samples, and is only evident (p<0.05) among the wetland soil samples (Table 3). In contrast, a strong positive relation of THg and SOM is evident in the B horizon soil samples at Fishing Brook, and is consistent throughout all three landscape types. At MC, THg and SOM were significantly positively related in all landscape types except for the Oa/A horizon wetland samples, and in all landscape types except for the riparian in B horizon samples (Table 3).

**Figure 5:**
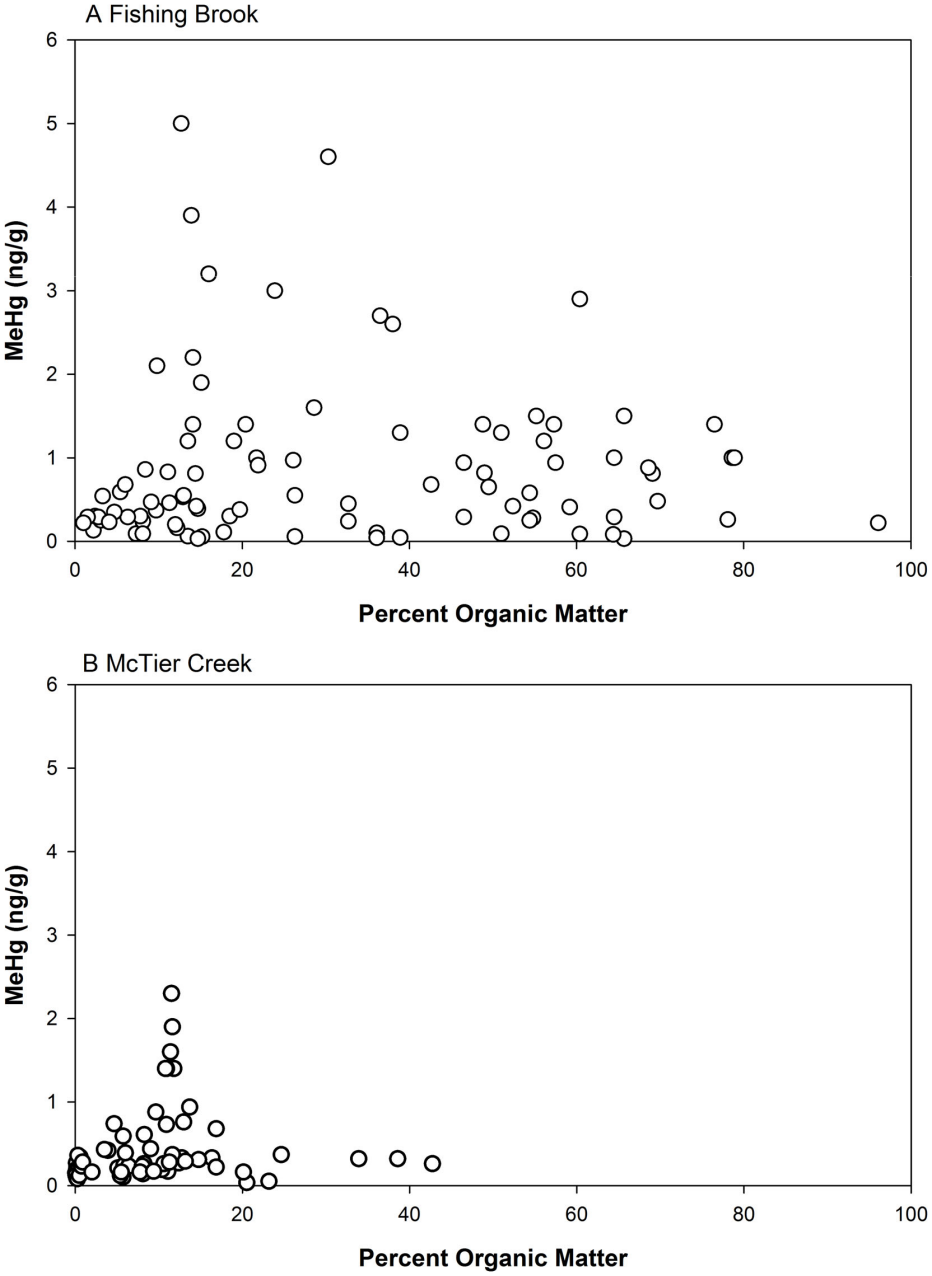
Methyl Hg and percent organic matter for all soil samples collected in each watershed. Absence of a line in panel indicates that a significant linear regression relation was not evident. A Fishing Brook, B McTier Creek.

Overall, far fewer significant MeHg-SOM relations were evident in these data than were evident in the THg-SOM data. For all soil samples collected at each site, the relation between MeHg and SOM was not significant (Fig. 5). Among soil depths, there were no significant MeHg-SOM relations for any landscape type at either watershed (Table 3). In B horizon soil samples by contrast, MeHg was positively related to SOM among all samples for both watersheds. However, only the wetland soil at FB showed a significant MeHg-SOM relation, whereas the relation at MC was of marginal (p = 0.094) significance.

### Hg Storage in Soil of the Two Watersheds

**Figure 6:**
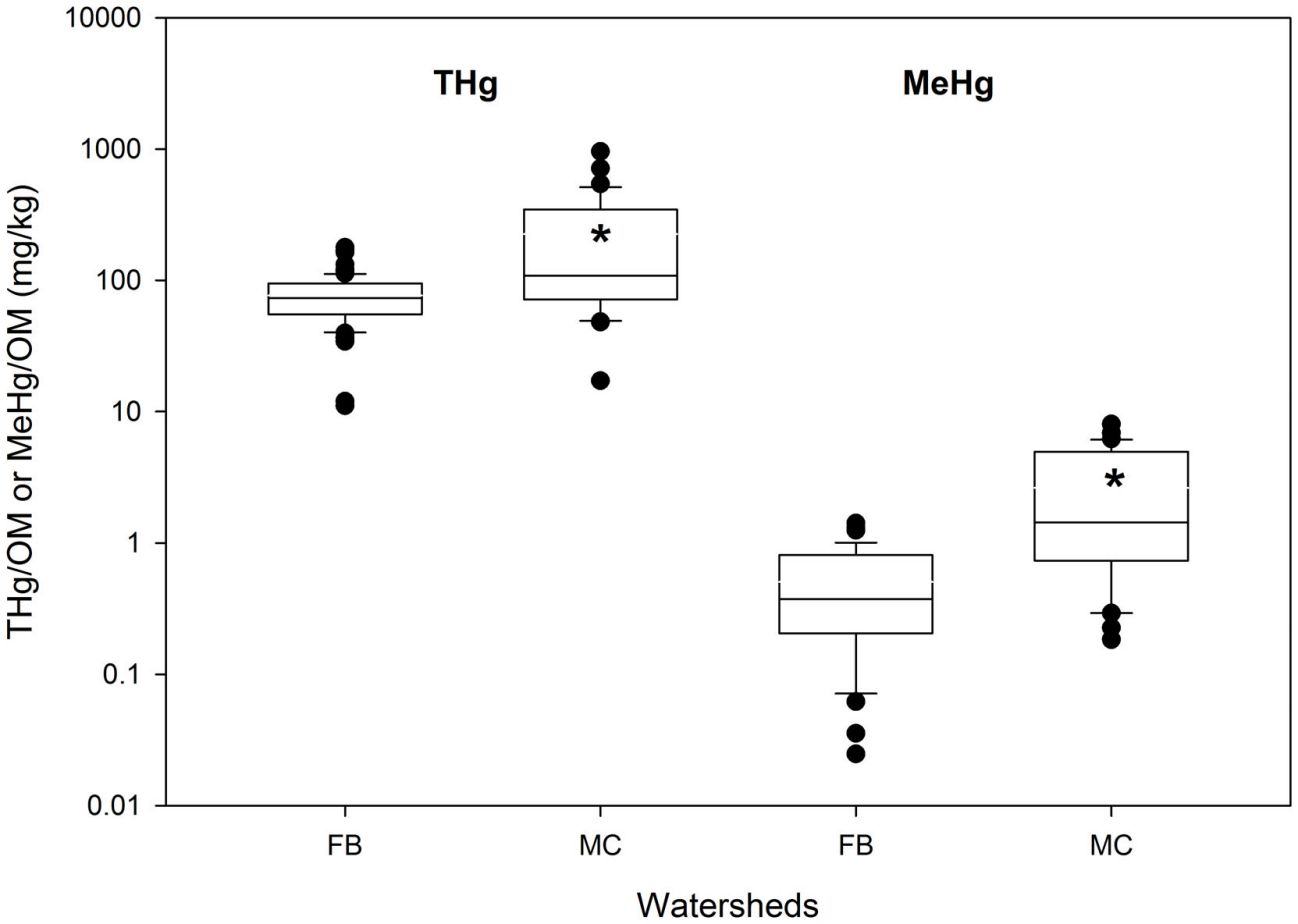
THg/OM and MeHg/OM based on estimates of storage in soil at each watershed. An asterisk in the box indicates the watershed with significantly greater values for each of the quantities shown.

Stores of THg in FB were two- to four-fold greater (p<0.05) than those in MC across all landscapes types (Table 4). In contrast, there were no significant differences in MeHg stores between these watersheds among any of the landscape types, although MeHg stores in MC were marginally greater (p = 0.058) than those in FB. Stores of SOM were about eight-fold greater overall in FB than in MC, and these differences were highly significant (p<0.001) across all landscape types. These strong differences in SOM storage among the two watersheds suggest that THg and MeHg are more efficiently stored as a function of available SOM in MC than in FB. This suggestion is confirmed by noting that median values of THg/SOM were about three-fold greater in MC than in FB, and median values of MeHg/SOM were nearly four-fold greater in MC than in FB (p<0.001; Fig. 6).

**Table 4:** Median estimated areally-normalized storage of total Hg, methyl Hg, and organic matter to a depth of 40 cm in the Fishing Brook, NY and McTier Creek, SC watersheds.

Site/Landscape Position	THg (µg/m^2^)	MeHg (µg/m^2^)	Organic Matter (kg/m^2^)
***Fishing Brook***			
All^1^	**2570**	8.4	**38.8**
Upland	**2540**	7.3	**39.8**
Riparian	**3020**	11.1	**33.5**
Wetland	**2220**	15.1	**37.2**
***McTier Creek***			
All^2^	690	10.3	4.7
Upland	650	11.0	2.6
Riparian	760	5.1	13.2
Wetland	980	11.1	12.7

Note – bold font indicates median value was significantly greater (p<0.05) in site-to-site Mann-Whitney pairwise comparison tests done for all samples collected and for each landscape type.

1 Represents 96.6% of watershed area excluding land cover not sampled in study (open water+developed land = 3.4%).

2 Represents 79.3% of watershed area excluding land cover not sampled in study (open water+developed land+agricultural land = 20.7%).

The differences in soil stores of THg, MeHg, and SOM among landscape types within each watershed were less distinct than those identified between the watersheds (Table 4), but some differences were noted (values not shown). THg stores were statistically indistinguishable among landscape types in both watersheds. Similarly, there was no difference in MeHg soil stores among landscape types in MC. In contrast, a statistically significant difference in MeHg stores was identified in FB, with values decreasing in the order wetlands = riparian>uplands, according to pairwise comparisons. For SOM stores, no differences among landscape types were noted at FB, but these values differed across landscapes in MC, decreasing in the order uplands>wetlands = riparian.

## Discussion

### Comparisons with Data from Other Studies

Hg concentrations and estimates of soil storage in these two watersheds are broadly consistent with values previously reported for the US and globally for rural sites where atmospheric deposition is the dominant input to the landscape [3], [6], [61], [62]. THg concentrations in soil from rural areas reported previously generally range from 50 to 300 ng/g, with greater values generally found in soil with high SOM levels, particularly in shallower soil horizons where SOM is concentrated [6], [7], [15], consistent with the results reported here. Fewer soil data have been reported for the southeastern US in locations similar to the MC watershed; however, two studies report THg concentrations in rural southeastern US sandy soil of about 10 ng/g [7] and 30 ng/g [63], broadly consistent with the generally lower median Hg values at MC (A = 60 ng/g, B = 30 ng/g) than those of FB (Oa = 200 ng/g, B = 85 ng/g).

Few studies have previously reported MeHg data in soil; however, some have reported MeHg/THg, with values as high as 5% [14], but most <1% [4], [6]. Here, we reported values of about 0.5% at FB and close to 1% at MC, consistent with most studies reviewed by Grigal [6] and Obrist [4]. Our data do not show a consistent pattern in MeHg/THg with soil depth across these two watersheds and the three landscape types sampled, but there is a pronounced tendency for the greatest values of MeHg and MeHg/THg to occur in riparian and wetland soil within each watershed. This pattern is consistent with those reported previously in these two watersheds and in other settings in which wetlands and riparian areas have the highest MeHg concentrations in soil, soil water, and shallow groundwater, and are the dominant source of MeHg to nearby surface waters [14], [25], [37].

The estimates of THg storage in FB soil of about 2500 µg/m^2^ (to 40 cm depth) are within the range of most values reported previously in forested landscapes in the northeastern US [64], and lower than those reported in peat soil and peatlands [3], [6], [13], [65]. The FB THg soil stores are less than those in a nearby watershed in the western Adirondacks of New York [16], but these latter estimates included the Oi and Oe horizons that were not sampled in FB, and also extended to a depth of 1 m, whereas THg stores were estimated only to a depth of 40 cm at FB. The estimated THg soil stores in MC of about 700 µg/m^2^ are generally less than those reported in most previous studies, and at the low end of estimates of mean soil THg storage over large regions in the US and Canada [6], [66]. We are not aware of any previously published estimates of soil THg stores from uncontaminated sites in the US Coastal Plain other than that of Obrist [4] for a site near Gainesville, FL. Soil THg concentrations are about 200 ng/g in the Everglades [67], where the soil is rich in peat and not readily comparable to the sandy soil of the South Carolina Coastal Plain.

### Comparisons between these Two Watersheds

The study watersheds have been previously identified as Hg “hot spots”, and show little inter-watershed difference in THg and MeHg concentrations in surface waters, aquatic invertebrates, and fish [25], [26]. For example, median filtered THg and filtered MeHg concentrations were similarly about 2 ng/L and 0.1 ng/L, respectively at MC and FB during 2007–09 [68]. Although soil is the likely dominant immediate source of THg and MeHg to local streams and biota in these two watersheds (Hg in bottom sediment and direct atmospheric deposition are viewed as minor sources), there are large differences in THg concentrations and stores between these two locations, with about three-fold greater values in FB than in MC. As previously noted, however, there are large uncertainties in estimating chemical element storage in watersheds including accounting for rock content, which was not estimated in this study. Despite these uncertainties, we believe that the three-fold difference is greater than the uncertainties that arise in estimating Hg stores.

In contrast, differences in soil MeHg between these two watersheds show a more complex pattern. MeHg concentrations at FB were generally greater overall and in most landscape types than those at MC (though differences were not always statistically significant), but there was little difference in MeHg stores between the watersheds. Furthermore, MeHg/THg was generally greater in the MC watershed than in the FB watershed. These results are broadly consistent with previous studies that have shown increases in concentrations and stores of THg [4] and SOM [69] with increasing latitude in the US, but are inconsistent with previous observations that similarly showed soil MeHg concentrations and stores increasing with latitude [4].

The size of the soil THg pools relative to estimates of annual atmospheric deposition of Hg differs sharply at these two sites. The current soil THg pool is equivalent to about 400 years of stored Hg relative to the current annual atmospheric Hg deposition rate at Fishing Brook of 6.3 µg/m^2^/yr, whereas the THg pool at McTier Creek is equivalent to about 70 years of stored Hg relative to current annual Hg deposition of 9.9 µg/m^2^/yr [70], a more than 5-fold difference among these sites. In contrast, the same estimates for the soil MeHg pool suggests similar relative storage in these two watersheds (FB = 267 yrs, MC = 208 yrs, assuming MeHg deposition is 0.5% of THg deposition, [71]), though MeHg deposition rates are highly uncertain.

These findings suggest a conceptual model that includes the role of soil in the Hg cycle at these two locations. A much larger proportion of the atmospheric THg deposition that has historically fallen at the Adirondack site remains stored in watershed soil, consistent with the colder climate and larger SOM pool relative to the Coastal Plain. Thus, the soil in the FB watershed represents a large, persistent pool of THg in contrast to the soil of the MC watershed, which turns over more rapidly. Lower rates of annual evapotranspiration and higher annual streamflow in concert with the larger soil Hg pools in the Adirondack watershed imply the potential for greater annual stream THg yields at the more northern location, which is consistent with modeled estimates at these two sites during 2007–09 [70]. In the MC watershed, the rates of other loss processes such as gaseous emissions resulting from respiration of soil organic matter and volatilization of Hg^0^ [2] are necessarily much greater than those in the Adirondack watershed to account for the much smaller THg pool in the Coastal Plain soil. Furthermore, the weak relation of THg to SOM in the Adirondack watershed soil with high organic matter content suggests that SOM composition is highly heterogeneous, consistent with the diverse specific ultra-violet absorbance values of DOC and relatively weak relation of DOC concentrations and filtered THg concentrations measured in Fishing Brook [72]. These results also suggest the likelihood of excess Hg binding capacity in this northern soil [8], whereas the retention of THg in the southern soil appears to be more strongly dependent on available SOM, which may be limiting retention.

Greater net methylation efficiency is suggested at the southern Coastal Plain site because the pool sizes are similar among the two sites, the annual stream fluxes are greater at FB than at MC [70], and MeHg/THg is generally greater at MC. However, the role of demethylation rates, a potentially important factor not measured in this study, may also play an important role in mediating MeHg/THg ratios in soil [73]. Nonetheless, a greater proportion of the soil THg pool is likely converted to MeHg on an annual basis at the southern site, necessary to provide the similar MeHg concentrations in streams and aquatic biota observed among these two watersheds [25], [26]. We hypothesize that warmer soil temperatures in the southern Coastal Plain than in the Adirondacks is the primary driver of the apparent greater net methylation efficiency at MC. Other factors, however, such as differences in the vegetation and soil between these sites may also play a role.

A potentially important caveat to these results and the conclusions drawn is that soils were sampled only once in each watershed. Little is known from the literature about the extent to which soil concentrations and stores vary seasonally. Our observations of THg and MeHg in springs, streams, and piezometers indicate that aqueous concentrations can vary two- to three-fold throughout the year in these two watersheds [25], and a similar magnitude of variation is possible in soils, though the large size of the stores would be expected to dampen this variation relative to that in solution.

### Differences Across Landscape Types

Previously published findings from these two watersheds and nearby indicate that wetlands and riparian areas are major sources of THg and MeHg to local streams, and flow paths that transport Hg species from these landscape settings to streams are the dominant control on the spatial and temporal variations in concentrations and loads of these Hg species [25], [27], [37], [74]. When soil concentrations and stores of THg and MeHg are compared among landscape types, including low Hg-yielding uplands, the differences are not as striking as those observed in surface waters. There were no significant landscape differences in THg concentrations or stores in FB soil, but MeHg values were greater in wetland soil, particularly when compared with those of upland soil. MC had significantly greater THg and MeHg concentrations in wetlands than those in riparian areas and uplands, but the stores of these Hg species were not significantly different among the three landscape types. The data from the current study are consistent with a prominent role for factors other than soil THg and MeHg concentrations and stores as principal controls on landscape variation in Hg cycling rates to streams. These factors include soil drainage characteristics, proximity to the stream channel, and the high water table and fluctuating redox processes (anoxic to oxic) in the relatively flatter wetland and riparian terrain. In particular, differences in the hydrological and biogeochemical processes that affect the concentrations and relative fluxes of dissolved organic matter (DOM) as well as the relative aromatic vs. aliphatic character of this DOM appear to be the principal controls on landscape variation in THg and MeHg concentrations and loads in streams in these two geographic settings [25], [27], [72].

## Conclusions

The results of this study show that two watersheds in different geomorphic and climatic regions with similar THg and MeHg concentrations in surface water and aquatic biota have greatly different concentrations and stores of THg in soil. THg concentrations and stores (to a depth of 40 cm) were about three-fold greater in the Adirondack watershed than those of the Coastal Plain watershed. Despite somewhat higher MeHg concentrations in the Adirondack soil, MeHg stores did not differ significantly among these two locations, although MeHg/THg was generally greater at the Coastal Plain site. These results are consistent with greater annual rates of net methylation and greater methylation efficiency in the soil of the warmer southern site. The greater soil stores of THg in the Adirondack watershed are consistent with an eight-fold greater store of SOM than that of the Coastal Plain watershed, though the relation of THg to SOM was weak in the Adirondacks, suggesting heterogeneous SOM and an excess of Hg-binding thiol groups relative to the net Hg supply. Furthermore, these results indicate that a greater proportion of historical atmospheric Hg deposition is stored in the Adirondack soil and that THg has about a five-fold greater soil turnover time than in the southern Coastal Plain soil. Comparison with known rates of atmospheric Hg deposition and stream export implies that gaseous Hg emissions from the Coastal Plain soil are necessarily much greater than those of Adirondack soil. These results raise questions about the ultimate fate of the Hg stored in the soil of these two sites. Past work has indicated residence times of several decades to several hundred years for atmospherically-deposited Hg, consistent with the results reported here. The overall greater store of THg in the Adirondacks suggests that declines in THg concentrations in stream water of this region will be more gradual and prolonged as future Hg deposition declines and comparatively sharper and less prolonged in Coastal Plain streams.
